# Network analysis of eating disorder and depression symptoms among university students in the late stage of COVID-19 pandemic in China

**DOI:** 10.3389/fnut.2023.1176076

**Published:** 2023-05-25

**Authors:** Weixin Yang, Dongmei Xiao, Yuchen Shi, Tianyuan Dong, Peng Xiong

**Affiliations:** ^1^Department of Public Health and Preventive Medicine, School of Medicine, Jinan University, Guangzhou, China; ^2^School of Medicine, Jinan University, Guangzhou, China; ^3^Capital University of Economics and Business, Beijing, China

**Keywords:** social network analysis, eating disorder, depression, COVID-19, Chinese students

## Abstract

**Background:**

Eating disorders (EDs) and depression are common in university students, especially during the COVID-19 pandemic. The aim of this study was to elucidate characteristics of EDs and depression symptoms networks among Chinese university students in the later stage of the COVID-19 pandemic in China.

**Methods:**

A total of 929 university students completed the SCOFF questionnaire measuring EDs and Patient Health Questionnaire with 9 items (PHQ-9) measuring depression in Guangzhou, China. The network model was applied to identify central symptoms, bridge symptoms, and important connections between SCOFF and PHQ-9 using R studio. The subgroup analyses of both genders in medical and non-medical students were further explored.

**Results:**

In the networks of the whole sample, central symptoms included “Loss of control over eating” (EDs) and “Appetite changes” (depression). The bridge connections were between “Loss of control over eating” (EDs) and “Appetite changes” (depression), between “Deliberate vomiting” (EDs) and “Thoughts of death” (depression). “Appetite changes” (depression) and “Feeling of worthlessness” (depression) were central symptoms in both subgroups of medical and non-medical students. “Fatigue” (depression) was the central symptom in the female and medical students group. The edge between “Loss of control over eating” (EDs) and “Appetite changes” (depression) acted as a bridge in all subgroups.

**Conclusion:**

Social network approaches offered promising ways of further understanding the association between EDs and depression among university students during the pandemic of COVID-19 in China. Investigations targeting central and bridge symptoms would help to develop effective treatments for both EDs and depression for this population.

## Introduction

The global pandemic caused by the coronavirus disease 2019 (COVID-19) since December 2019 has had significant repercussions, disrupting daily life and causing physical and mental health concerns. Alongside quarantine and isolation measures (1), this crisis has led to an upsurge in psychological distress, such as eating disorders (EDs) and depression, among the general population (2). EDs are disabling, potentially lethal, and expensive mental health conditions that significantly compromise physical health and disrupt psychosocial functioning (3). Negative attitudes toward weight, body shape, and eating habits play a crucial role in the development and maintenance of EDs (3). EDs were prevalent among university students, as evidenced by previous studies (4–6). A recent meta-analysis of 15 cross-sectional studies with 41,956 Chinese university students reported a depression prevalence rate of 27% during the COVID-19 pandemic (7).

EDs and depression often co-occur, as evidenced by research on both clinical and non-clinical populations. Specifically, non-clinical studies have shown that students at risk for EDs tend to have higher depression scores than their counterparts without EDs risk (8), while a clinical study found that almost 20% of EDs patients had comorbid major depression and nearly half reported clinically significant depressive symptoms (9). A survey conducted during the COVID-19 pandemic among Italian college students also revealed a positive association between psychological problems such as depression, anxiety, and tension, and EDs symptoms such as interoceptive awareness and binge eating behaviors, regardless of gender differences (10).

EDs and depression have a bi-directional relationship. In non-clinical samples, eating pathology significantly predicted depression, possibly due to the failure to achieve an idealized physical ideal or weight gain caused by binge eating (11). Depression, on the other hand, could predict the development of eating pathology as a compensatory mechanism to reduce negative affect (12). Notably, depression has different effects on the development of EDs in male and female adolescents. For females, depression has a direct impact on body dissatisfaction and EDs symptoms, while for males, it has a greater moderating effect on sociocultural influences (13). Low self-esteem and self-directedness are also linked to the development of EDs via depression (13). The “common-cause” model posits that cognitive inefficiency underlies both depression and EDs, leading to their comorbidity (14).

Network analysis is an increasingly utilized model for exploring comorbidity between disorders in psychopathology research (15). In network approaches, disorders are viewed as systems of causally interconnected symptoms rather than being solely dependent on latent variables (15), which indicates that these problems interact with and influence each other (16–18). Therefore, these systems can be represented, analyzed, and studied in their full complexity. Moreover, network modeling has the added philosophical benefit of rejecting the unrealistic notion that symptoms of a single disorder have a singular causal background (15). According to network theory, the nodes in the network represent symptoms of disorders. Central nodes are those that are more important than others within the framework (19). Important symptoms in a disorder could be detected by computing three centrality indices, namely strength (measuring direct connections between symptoms), closeness (measuring indirect connections), and betweenness (measuring the importance of a symptom in the average path between two other symptoms) (19). Identifying these key symptoms can aid in the selection of ideal treatment targets and inform clinical treatment decisions (18). In addition, edges, which represent relationships between two symptoms, are another vital element in the network. These edges vary in terms of their edge weights or strength of linkages (19). Among all nodes, bridge symptoms identified through bridge centrality computation are considered potential pathways through the comorbidity of two disorders (17). The identification of bridge symptoms is valuable in making treatment of comorbid disorders more targeted and problem-focused.

Several studies have utilized network analysis to examine EDs both in clinical and non-clinical samples. Within non-clinical samples, cognitive symptoms related to shape and weight concern, such as “shape overvaluation,” “weight dissatisfaction,” and “desiring weight loss,” emerged as highly central symptoms in the EDs network (20–22) and some clinical samples (23, 24). However, rather than “body dissatisfaction” and “drive for thinness”, “interoceptive awareness” and “ineffectiveness” were found to be central to the EDs networks in other clinical samples (25). Network analysis has also been used to examine depression in different populations. In adolescents, core symptoms in depression networks included “sadness,” “self-hatred,” “loneliness,” “self-deprecation,” and “feeling like a failure” (26–28). During the COVID-19 pandemic, “loss of energy,” “psychomotor problems,” and “guilt feelings” were identified as the three central symptoms in the depression network in the community in Macao (29).

Existing research on EDs and depression using network analysis has primarily focused on early adolescent samples. In the clinical samples, certain physical symptoms such as “restlessness,” “low self-esteem,” and “feeling overwhelmed” have been identified as bridge symptoms linking EDs to depression (24). In non-clinical adolescent samples, core symptoms of EDs have been identified as the desire to lose weight (30, 31), dissatisfaction with shape and weight (31), and preoccupation with shape or weight (31). On the other hand, core depressive symptoms of depression in these non-clinical samples included feeling “depressed,” “lonely,” and experiencing “low energy” (31). Furthermore, additional bridge symptoms connecting EDs and depression in these non-clinical samples include “feeling like a failure” (30), “irritable,” (31) “social eating,” (31) and “depressed” (31). However, the generalizability of the above-mentioned results to university student populations is uncertain, especially during the COVID-19 pandemic when a significant increase in these disorders has been reported (32, 33). It is worth noting that female university students have been found to be at higher risk of developing EDs (8, 34).

A deeper understanding of the intricate relationship between EDs and depression could greatly improve the efficacy of interventions in educational and clinical settings. However, there is a notable lack of network analysis examining the symptoms of EDs and depression specifically during the COVID-19 pandemic. Given the unique characteristics of Chinese university students, it is crucial to investigate the underlying mechanisms that contribute to the co-occurrence of EDs and depression. To the best of our knowledge, this study represents one of the pioneering efforts to explore the comorbidity between EDs and depression among Chinese university students, particularly within the context of COVID-19 pandemic.

Therefore, in this study, we approached social network analysis to investigate the central and bridge symptoms between EDs and depression symptoms among Chinese university students during the later stage of the COVID-19 pandemic. We also examined the potential differences in subgroups of male and female students, as well as medical and non-medical students.

## Methods

### Participants and procedure

A cross-sectional survey using structured questionnaires was performed among university students in Guangzhou, China. The data was collected from October 2020 to December 2020, when COVID-19 was under control and alleviated in China. An online questionnaire (wjx.cn, which is one of the most popular online survey platforms in China) was distributed to university students via WeChat groups and WhatsApp messaging application with the snowball sampling method. A total of 929 participants completed the survey. The present study was approved by the ethics committee of Jinan University, and all participants provided e-written informed consents prior to participation. Participant characteristics are provided in Table 1.

**Table 1:** Participant characteristics (*N* = 931).

		*N*	Mean	SD	Range
Age (years)	Total	927	21.03	2.614	17–50
	Males	298	21.55	3.427	17–50
	Females	629	20.79	2.082	17–36
	Medical students	596	20.68	2.273	17–36
	Other major	331	21.66	3.040	17–50
BMI (kg/m^2^)	Total	927	21.17	3.591	13.79–42.34
	Males	298	22.71	3.661	15.78–34.33
	Females	629	20.43	3.318	13.79–42.34
	Medical students	596	21.44	3.619	13.79–37.59
	Other major	331	20.67	3.49	14.68–42.34

SD, Standard Deviations.

### Measurements

#### Demographics information

Demographic information was collected, including age, gender, major, educational level, and BMI $(\mathrm{Formula}\;:\mathrm{BMI}=\frac{\mathrm{weight}\;\left(\mathrm{kg}\right)}{{\mathrm{height}}^{2}\left(m^{2}\right)};\mathrm{kg}/m^{2}).$

#### Eating disorders symptoms

Eating disorder symptoms were assessed with the SCOFF questionnaire (35). SCOFF is an acronym derived from the first letter of the focus word in each of the five eating-related items that focus on the core features of anorexia nervosa and bulimia nervosa. The five SCOFF questions were: “Do you make yourself Sick because you feel uncomfortably full?,” “Do you worry you have lost Control over how much you eat?,” “Have you recently lost more than One stone in a 3-month period?,” “Do you believe yourself to be Fat when others say you are too thin?,” and “Would you say that Food dominates your life?.” Each item rates on a dichotomous score of yes (“1”) or no (“0”). The scores on each item were then added up, resulting in a total score ranging from 0 to 5. A score of ≥2 was considered indicative of anorexia nervosa or bulimia. The SCOFF questionnaire has been validated in the Chinese population (36) and was used in the China Health and Nutrition Survey (CHNS) in 2015 (37). In the present study, the Cronbach’s alpha of this scale was 0.7, indicating good reliability.

#### Depression symptoms

The individual’s depression symptoms were measured using the Patient Health Questionnaire with 9 items (PHQ-9) (38), which anchors to the past 2 weeks. Each item is rated on a four-point scale ranging from 0 (not at all) to 3 (nearly every day), with a higher score indicating a greater frequency and intensity of depression symptoms. An example item is “How often have you been bothered by feeling down, depressed, or hopeless?.” The total score ranges from 0 to 27, with higher scores representing more severe depression symptoms. The Chinese version of the PHQ-9 has been found to have good psychometric properties among university students in previous studies (39, 40). In our sample, the Cronbach alpha was 0.91, suggesting excellent reliability.

### Data analysis

Descriptive statistics were analyzed for the demographic variables and scores of questionnaires and scales, using SPSS 23.0. Continuous variables were presented with means, standard deviations (SDs), and ranges. Network models depicting the structure of EDs and depression symptoms and interactions between them were computed using R studio. As all consenting participants were prompted to complete all items, there was no missing data.

We constructed separate network models for EDs and depression items in five samples: the entire sample, as well as subgroups of male and female students, and medical and non-medical students.

Visualization of the network model was generated using the *qgraph* package in R (41). The graphic LASSO method was used to construct a network of regularized partial correlations between nodes (19). Thinker lines represent stronger relationships between symptoms. Solid lines denote positive relationships and dashed lines denote negative relationships.

The stability and accuracy of each network model were estimated using the *bootnet* package in R, with 1,000 case-dropping bootstraps. Significant differences between edge weights were tested with a 95% confidence interval. To assess the strength centrality index, a correlation stability coefficient (CS-coefficient) was used (19), with a recommend value over 0.25 and preferably greater than 0.5 (19). The *goldbricker* function in R was used to identify nodes measuring the same underlying construct, with a threshold set at 0.25. All pairs of nodes falling below this threshold were considered “bad pairs.”

We calculated and visualized centrality indices (e.g., strength, closeness, betweenness) of nodes using the *centralityPlot* function in the *bootnet* package in R (19). Strength centrality was used to identify the most central symptoms, as it has been found to be the most reliable measure of centrality (42). Centrality difference-tests were conducted via the *bootnet* package in R to assess significant differences in symptoms strength (19). To examine comorbidity between the two disorders, bridge symptoms were identified using the *bridge* function in the *networktools* package in R, which allowed us to identify which items were most interconnected across EDs and depression symptoms (43).

## Result

### Network structures of eating disorders and depressive symptoms

**Figure 1:**
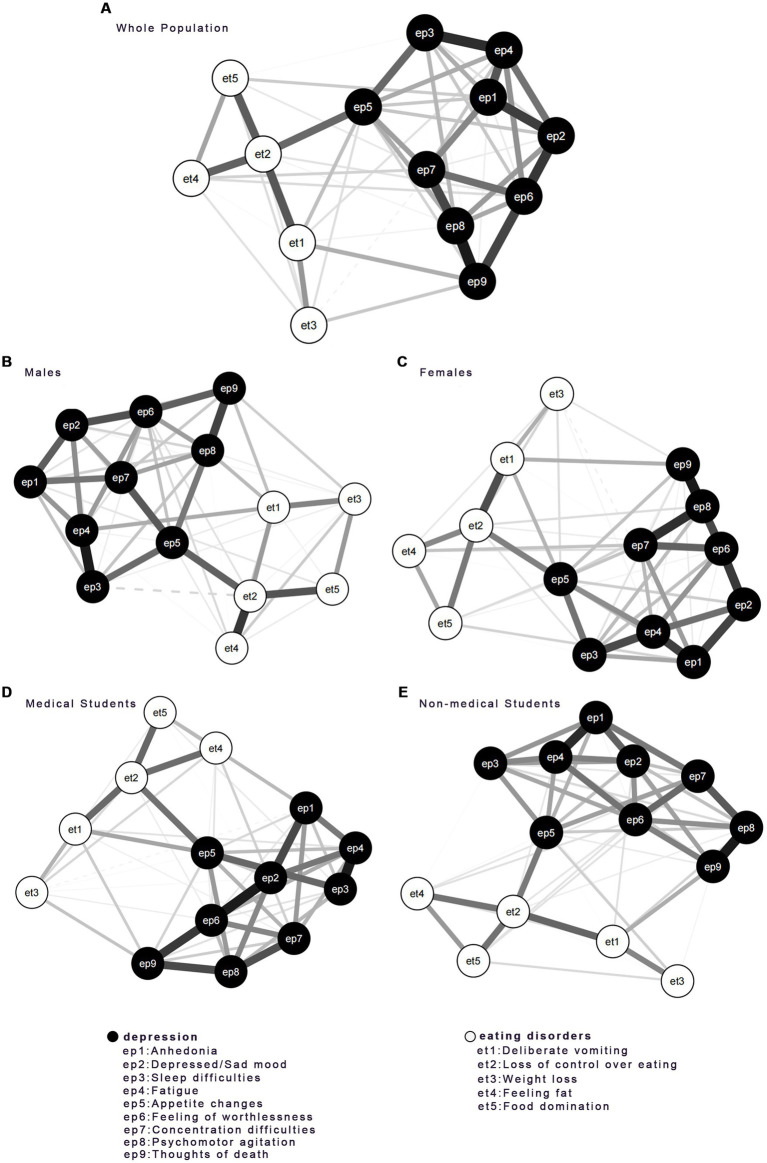
Networks of EDs and Depression for **(A)** the whole population, **(B)** males, **(C)** females, **(D)** medical students, and **(E)** non-medical students. EDs community was shown in white and depression items were shown in black. The thickness of the line depicted edge strength.

The networks displayed in Figure 1 showed the relationships between EDs and depression symptoms for the whole population, subgroups of male and female students, and medical and non-medical students. Table 2 showed the corresponding symptoms of each node in Figure 1 and reported the SDs and means of the EDs and depression symptoms scores.

**Table 2:** Item, item content, item abbreviations, means and standard deviations for SCOFF and PHQ-9 items.

Item	Item content	Item abbreviation	*M*	SD
et1	Sickness on feeling full	Deliberate vomiting	1.16	0.36
et2	Lose control over eating	Loss of control over eating	1.30	0.46
et3	Lose weight	Weight loss	1.12	0.32
et4	Thoughts of being fat	Feeling fat	1.38	0.48
et5	Food domination	Food domination	1.40	0.49
ep1	Anhedonia	Anhedonia	1.94	0.88
ep2	Depressed or sad mood	Sad mood	1.85	0.83
ep3	Sleep difficulties	Sleep	1.88	0.95
ep4	Fatigue	Fatigue	2.09	0.89
ep5	Appetite changes	Appetite	1.77	0.90
ep6	Feeling of worthlessness	Worthless	1.76	0.90
ep7	Concentration difficulties	Concentration	1.92	0.93
ep8	Psychomotor agitation/retardation	Motor	1.53	0.83
ep9	Thoughts of death	Death	1.40	0.77

M, Mean; SD, Standard Deviations.

The network for the whole population showed the strongest edges were found within the inter-community in EDs and depression. In the EDs community, they were et2 (Loss of control over eating) and et4 (Feeling fat), et2 (Loss of control over eating) and et5 (Food domination), et2 (Loss of control over eating) and et1 (deliberate vomiting). In the depression community, they were ep8 (Motor) and ep9 (Thoughts of death), ep6 (Worthless) and ep2 (Sad mood), ep3 (Sleep) and ep4 (Fatigue), which were the most positive correlations.

However, the symptoms significantly connected to et2 (Loss of control overeating) varied among the four subgroups. Among male students, et4 (Feeling fat) and et5 (Food domination) were directly linked to et2 (Loss of control over eating). Among female students, et1 (Deliberate vomiting) was directly linked to et2 (Loss of control overeating). For both medical students and non-medical students, et1 (Deliberate vomiting) and et4 (Feeling fat) were directly linked to et2 (Loss of control over eating). The item of et1 (Deliberate vomiting) was also intensely linked to et5 (food domination) in medical students.

The connections between depression symptoms showed significant differences among the four subgroups. Among male students, ep8 (Motor) was directly linked to ep9 (Thoughts of death), while ep3 (Sleep) was directly linked to ep4 (Fatigue). Among female students, ep7 (Concentration) and ep9 (Thoughts of death) were strongly linked to ep8 (Motor). Among medical students, ep2 (Sad mood) and ep9 (Thoughts of death) were intensely linked to ep6 (Worthless), while ep3 (Sleep) was linked to ep4 (Fatigue). For non-medical students, ep8 (Motor) was intensely linked to ep9 (Thoughts of death) while ep1 (Anhedonia) was linked to ep4 (Fatigue).

### Centrality comparison between nodes

**Figure 2:**
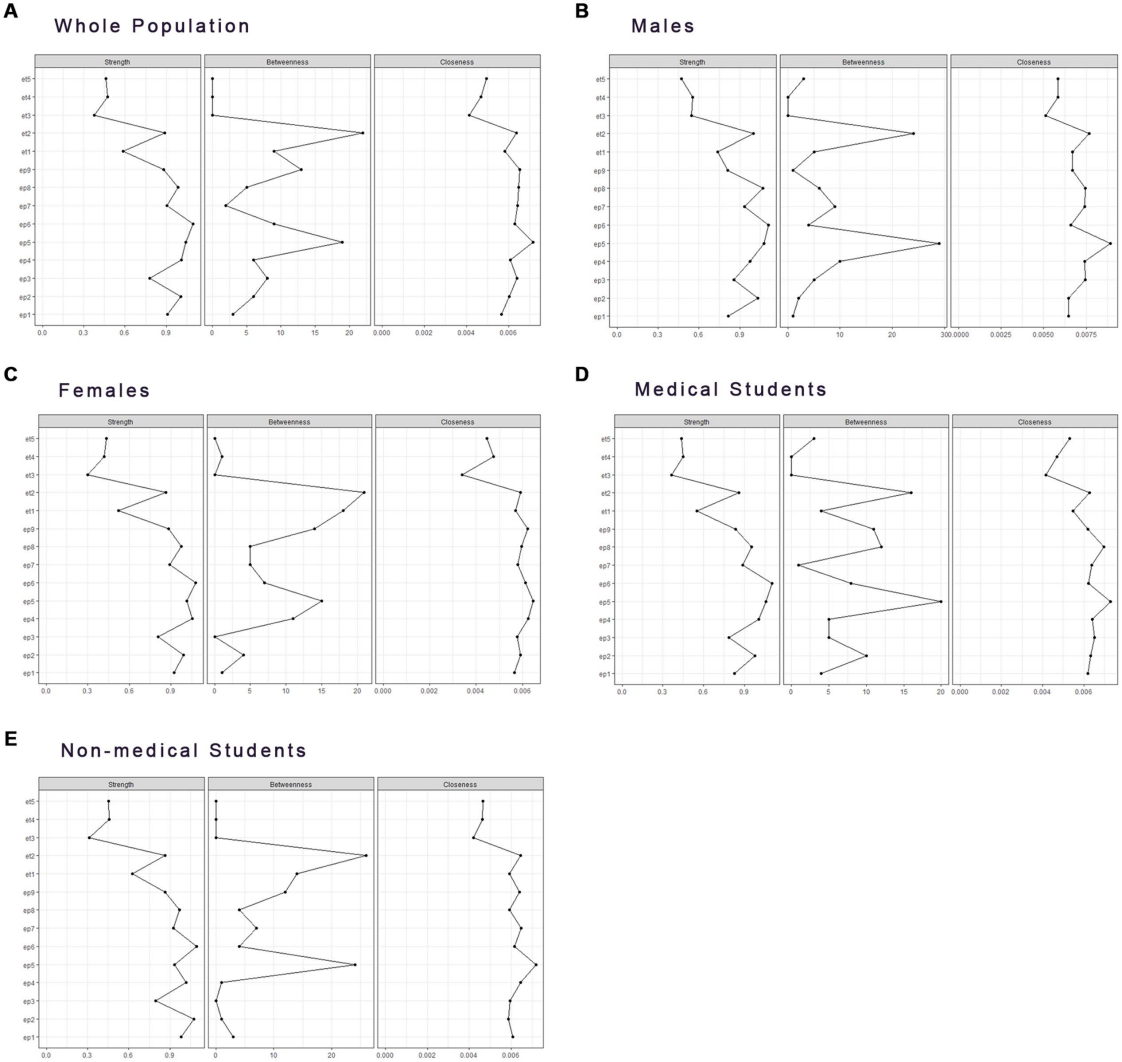
Centrality index of networks between EDs and Depression for **(A)** the whole population, **(B)** males, **(C)** females, **(D)** medical students, and **(E)** non-medical students.

Regarding centrality analysis (Figure 2), we evaluated the importance of each node based on its calculated scores. In the EDs networks, et2 (Lose control over eating) was consistently identified as the most central symptom in terms of centrality strength, betweenness, and closeness across all five networks. In addition, only the whole sample and female students subgroups’ networks had the second-highest betweenness centrality symptom—et1 (Sickness on feeling full), compared with other groups.

Among depression symptoms, ep5 (Appetite changes) was the most central node in terms of centrality strength in the network of the whole sample, as well as having the highest closeness and betweenness in four subgroups’ networks.

Other significant nodes varied among gender and major subgroups’ networks. In both gender subgroups’ networks, ep8 (Psychomotor agitation/retardation) had the highest strength in males, and ep4 (Fatigue) had the highest strength in females. Regarding networks of different majors, both medical students’ and non-medical students’ networks had ep5 (Appetite changes) and ep6 (Feeling of worthlessness) as the most central nodes in terms of strength. Additionally, in the medical students’ network, ep4 (Fatigue) had the second-highest strength, while ep8 (Psychomotor agitation/retardation) had the second-highest closeness and betweenness.

### Stability of centrality indices and accuracy of nodes

The strength stability of all networks with 95% confidence intervals via bootstrap was satisfactory (Supplementary Figures 1–5), the total group had a CS (cor = 0.7) of 0.75, the female and medical students had a CS (cor = 0.7) of 0.749, and the male and non-medical students had a CS (cor = 0.7) of 0.595. The *‘goldbricker’* function in R studio was employed to evaluate the bootstrap difference of each paired node, discovering no “bad pairs.” (Supplementary Figures 6–10).

### Edge comparisons and bridge symptoms identifications

**Figure 3:**
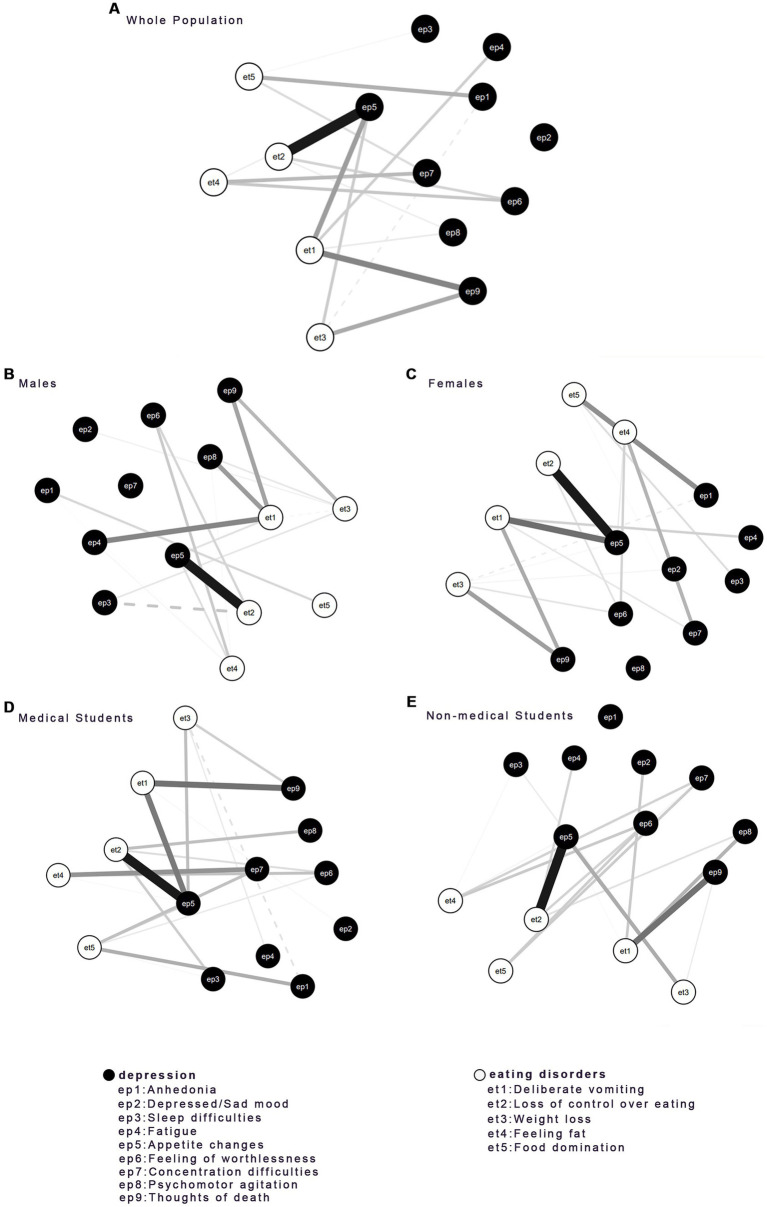
Bridge connection between EDs and depression disorder of all groups included **(A)** the whole population, **(B)** males, **(C)** females, **(D)** medical students, and **(E)** non-medical students. Solid lines represent positive connections, and dashed lines represent negative connections.

The network bridge connections were displayed in Figure 3. The edge between et2 (Loss of control over eating) and ep5 (Appetite changes) was the strongest bridge in all networks of EDs and depression. The edge weight was 0.2050 in the network of the whole sample, 0.2228 in the male group, 0.1613 in the female group, 0.1886 in the medical students’ group, and 0.1782 in the non-medical students’ group. Other bridge connections were: et1 (Deliberate vomiting) and ep9 (Thoughts of death) in the whole sample, edge weight = 0.1006; et1 and ep4 (Fatigue) in the male group, edge weight = 0.1174; et1 (Deliberate vomiting) and ep8 (Psychomotor agitation/retardation) in the male group, edge weight = 0.0976; et1 (Deliberate vomiting) and ep9 (Thoughts of death) in the female group, edge weight = 0.0996; et1 (Deliberate vomiting) and ep5 (Appetite changes) in the female group, edge weight = 0.0934; et1 (Deliberate vomiting) and ep5 (Appetite changes) in the medical students group, edge weight = 0.1107; et1 (Deliberate vomiting) and ep9 (Thoughts of death) in the non-medical students group, edge weight = 0.1095.

**Figure 4:**
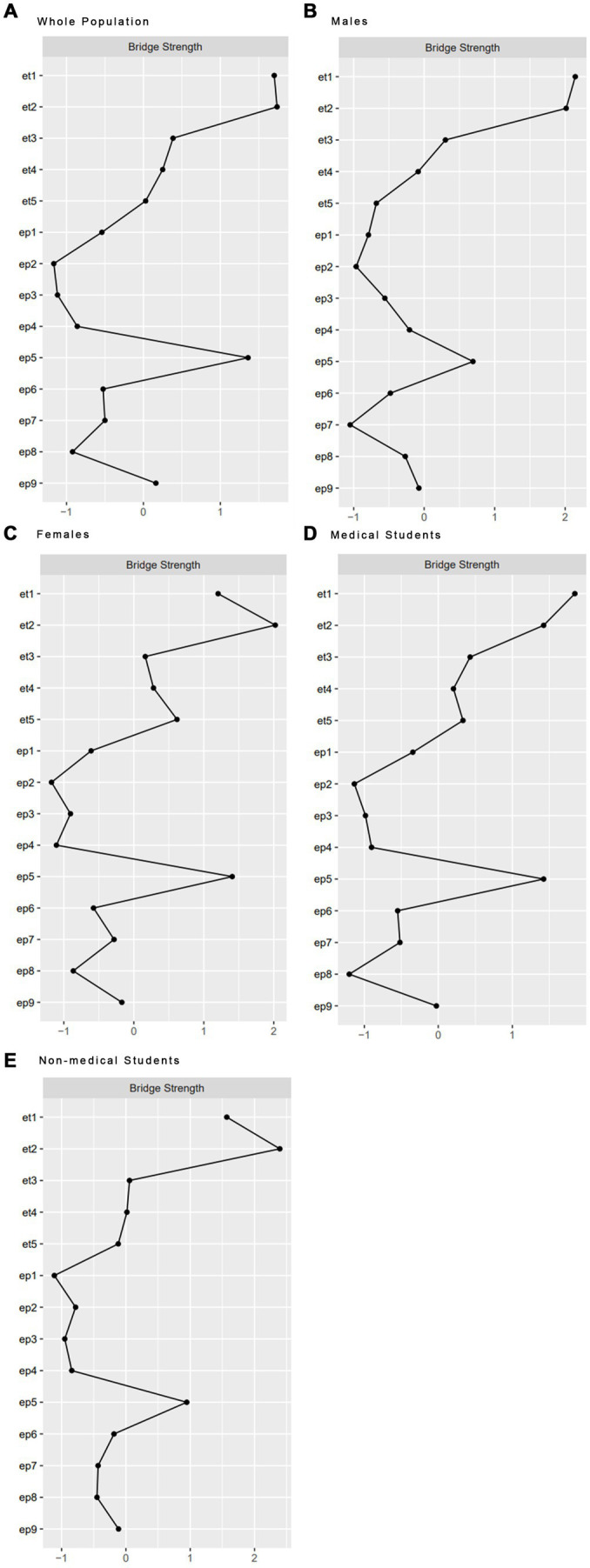
Bridge strength of all groups between EDs and Depression included **(A)** the whole population, **(B)** males, **(C)** females, **(D)** medical students, and **(E)** non-medical students.

Bridge symptoms elucidated the associations between EDs and depression (see Figure 4). The bridge symptoms in all groups, et2 (Loss of control over eating) (EDs) and ep5 (Appetite) (depression), might be efficacious targets in the prevention and treatment of the comorbidity of EDs and depression for university students with different genders and majors. Moreover, there was a variance in bridge symptoms across different groups. Ep6 (Feeling of worthlessness) (depression) served as a bridge symptom in the network of the whole sample. Et1 (Deliberate vomiting) (EDs) was the bridge symptom in the medical student group and the male group.

## Discussion

To the best of our knowledge, this is the first network analytic study of the comorbidity between EDs and depression among Chinese university students in the later stage of the COVID-19 pandemic in China. Through our analysis, we identified several crucial symptoms and edges, which offer potential targets and pathways for interventions. The observed comorbidity between EDs and depression partially aligns with the findings from a network analysis conducted among Chilean university students (44), which additionally highlighted the central role of physical anxiety symptoms in EDs.

### Bridge symptoms

Across the network of EDs and depression in the overall sample, we detected three key bridge symptoms, namely “Loss of control over eating” (EDs), “Appetite changes” (depression), and “Feeling of worthlessness” (depression). Among them, “Loss of control over eating” and “Appetite changes” also acted as bridge symptoms in the networks of subgroups of male, female, medical, and non-medical students, which corroborated their central roles in the link between EDs and depression. This result was consistent with the prior research exploring the associations between fear of losing control over eating and depressive symptoms among young adults in the United States (45). Individuals with greater endorsement of depression symptoms were more likely to lose control over eating. It has been reported that the odds of overeating increased during the COVID-19 pandemic (46). Emotional eating refers to the tendency to overeat as a reaction to negative feelings or stress. Negative feelings such as distress and depression could be a leading cause of the insurgency of emotional hunger (47). Factors of isolation, lack of stimuli, and food routines changing, which emerged and worsened during the lockdown of COVID-19, might influence people’s ability to control their eating habits (48). Such changes, including overeating, could increase students’ depression symptoms due to the worries about their health and fitness. Early identification and intervention of cognitive features like “Loss of control over eating” may therefore help reduce the risk of developing EDs and depression.

The depressive symptom of “Feeling of worthlessness” was a bridge symptom between EDs and depression, which was consistent with the findings in a previous study in Iranian adolescents and young adults (30), where “Feeling like a failure” showed the highest bridge centrality between depression and disordered eating. This was further supported by network analysis on mental health in patients with EDs (49). According to the model of schemas in the eating disorders posted by Waller et al., food restriction acted as the primary avoidance and bingeing acted as the secondary avoidance of the distress associated with negative self-brief (50). Thus, students feeling worthless were at high risk of developing disordered eating. Enhanced cognitive-behavioral therapy (CBT-E) has been developed to target feelings of worthlessness for EDs, which was confirmed in the previous findings (51).

For male students and medical students, it was observed that “Deliberate vomiting” (EDs) was a bridge symptom between EDs and depression. During the COVID-19 pandemic, Chinese male students were found to receive less social support when compared with females, which was associated with high levels of mental distress (e.g., depression, anxiety, and stress symptoms) (52). Females received more support due to their willingness to express their problems and stress, larger social networks (53), more sources to draw support, more satisfaction with friends, and the general acknowledgment that females need more protection than males (54). Additionally, staying up late playing games was more common in male students, which played a role in the development of irregular life rhythms, unhealthy eating habits, and gastrointestinal discomfort. Therefore, negative moods including depression and gastrointestinal distress both increased the odds of “Deliberate vomiting” in male students. To alleviate the level of EDs symptoms (e.g., vomiting) and depression symptoms in male students, more social support, targeting psychological interventions, and guidance on healthy life rhythm are needed. The COVID-19 pandemic has had a particularly strong impact on the educational integrity of medical programs. While lecture-based teaching was transitioned to an online format, clinical exposure and experiment learning were not as easily replicated. Difficulties in adjusting to the examination and curricular restructuring led to increased psychological distress, anxiety, and depression about the academic burden among medical students. Other sources of stress and depression include the possibility of being fast-tracked to the frontline or deployed to other areas of the health service, worries about their own health and well-being, and that of their family. Gastrointestinal discomfort caused by staying up late learning, coupled with high levels of depression, could make medical students more prone to vomit.

### EDs edges

Within the community of EDs symptoms, three positive edges were markedly higher than others, including the edge between “Loss of control over eating” and “Deliberate vomiting,” the edge between “Loss of control over eating” and “Feeling fat,” and the edge between “Loss of control over eating” and “Food domination.”

The previous study had shown “Feeling fat” to be a significant and independent predictor of dietary restraint and eating concerns (55), suggesting that students feeling fat are more likely to lose control of overeating. Additionally, body dissatisfaction (e.g., “feeling fat”), low self-esteem, and maladaptive social comparison, which might be common in student populations, had been indicated as disruptive psychological patterns common in EDs and unipolar depression (56). These findings highlighted the need for guidance to build a healthy body image in order to prevent EDs symptoms on campus. The unhealthy cult of slimming, which is now popular on social -media, needs to be addressed, as young adults are more vulnerable to information from media sources. Our findings also drew attention to the importance of exploring whether “feeling fat” can be used as an effective target in the treatment of EDs, and the development of standardized measures of “feeling fat.”

### Depression edges

Within the depression community, our result of a strong positive link between “Psychomotor agitation or retardation” and “Thoughts of death or self-harm” was consistent with results from other populations such as migrant Filipino domestic workers in China (57) and depressed children and adolescents in Hungary (58). Our results emphasized the importance of timely identification and cognitive interventions that address psychomotor agitation or retardation to block negative thoughts of death or self-harm as early among Chinese university students in the COVID-19 pandemic.

### EDs-depression pathways

The edge between “loss of control over eating” and “appetite changes” was a potential bridge between EDs and depression. The depression symptom “appetite changes” includes both poor appetite and overeating. The EDs symptom “loss of control over eating” measures the worry about losing control over eating. The overlap of items concerning overeating in both measures might be an important reason for the high positive correlation between the theses two symptoms. Future researchers exploring the association between depression and EDs symptoms should consider excluding overeating-related item in depression scales, or investigating overeating and poor appetite separately. The lockdown limited students’ ability to exercise at gyms or outdoors during the COVID-19 pandemic. Therefore, concerns about health and body image may be a motivating factor for the development of a restrained diet (59). Other risk factors, such as increased time with social media and the objectification of the ideal of thinness, could increase the likelihood of anorexia nervosa (59). Encouraging students to exercise at home may help to address some of their concerns during this specific stage, and it may also serve as a potential pathway to alleviate disordered eating and depression. Quarantine during the COVID-19 pandemic has caused feelings of isolation and distress among many individuals (60). Eating has played a significant role in alleviating stress and improving mood (61), and as a result, many people have turned to food as a source of comfort during the lockdown period (48). Emotional eating has become more common as people stayed at home alone (62, 63). This has increased the risk of symptoms related to eating disorders, such as “Loss of control over eating.” Therefore, interventions aimed at addressing symptoms of EDs among university students during the COVID-19 pandemic should target negative emotions such as anxiety, fear, and depression.

### Limitations

There are several limitations to this study that should be acknowledged. Firstly, the cross-sectional design of this study limited our ability to draw conclusions about the directionality of relationships between symptoms. Secondly, the current findings might not be generalizable to students who have experienced other types of disasters (e.g., earthquakes, typhoons). It is possible that the unique restrictions on living and studying environment due to the COVID-19 pandemic only partially account for the associations within symptom networks. Thirdly, cross-cultural differences may limit the generalizability of the network models to global student populations. Additionally, the study only used self-reported data, which could result in inaccurate reports for some participants who lack self-awareness of psychological symptoms. Lastly, the lack of clinical interviews for eating disorders and depression is a limitation of this study.

### Strengths

Despite the limitations, the current study also has several strengths. Firstly, the study included a large sample of university students who were known to have difficulties with EDs and depression impairments. This enabled us to provide reliable and valid evidence of the underlying relationships between these disorders, which could inform the development of interventions. To the best of our knowledge, most network analyses of EDs and depression focused on clinical samples, and the non-clinical research mainly focused on adolescents (31), with university students receiving less attention. Our study filled this gap. Furthermore, the instruments used to assess EDs and depression in our study were validated for use in Chinese students (36, 40), and the SCOFF had been proved to be a valid and reliable screening tool for EDs screening in written form (64). We asked all items in the scales, which allowed for more accurate network estimates. To minimize bias, we excluded the data from questionnaires that were completed too quickly (less than 120 s). Finally, the strength centrality indexes of all networks were highly stable, which values above 0.50 (19).

### Implications

Our study findings have important implications for future clinical practice. We found that the strongest connection in our network was between EDs symptom “Loss of control over eating” and depression symptom “Appetite changes.” Therefore, interventions aimed at improving these symptoms may be practical treatment approaches for university students experiencing these disorders during the COVID-19 pandemic. Targeting bridge symptoms through interventions may have the potential to improve symptoms transdiagnostically in individuals with comorbid EDs and depression. Specifically, the examination of variables such as “Loss of control over eating,” “Appetite changes,” and “Feeling of worthlessness” could serve as potential targets for reducing symptoms of both EDs and depression. It would be valuable for further investigations to explore the influences of these symptoms on university students during the pandemic, particularly focusing on the specific context of Chinese university students. Experimental studies could be designed to examine the effects of educational and social cues, as well as threats related to “Loss of control over eating,” “Appetite changes,” and “Feeling of worthlessness” among Chinese university students, in order to gain a better understanding of these factors and their impact on individuals in this population. By targeting and addressing these symptoms, interventions may hold promise in improving the overall well-being of Chinese university students facing EDs and depression. Additionally, further research on cross-cultural symptom networks of the association between mental problems (such as EDs and depression) could provide new insights. Longitudinal studies of the comorbidity could also help to understand the directionality, variability, and interaction of symptoms within the network over time.

## Data availability statement

The original contributions presented in the study are included in the article/Supplementary material, further inquiries can be directed to the corresponding author.

## Ethics statement

The studies involving human participants were reviewed and approved by the ethics committee of Jinan University. The patients/participants provided their written informed consent to participate in this study.

## Author contributions

WY and DX: methodology, interpretation, writing—original draft, and writing—review and editing. YS: methodology, data analysis and interpretation, and writing—original draft. TD: methodology, data analysis and interpretation, and writing—original draft. PX: conceptualization, data collection, methodology, writing—review and editing, supervision, project administration, and funding acquisition. All authors contributed to the article and approved the submitted version.

## Funding

This work was supported by “Guangzhou Basic and Applied Basic Research Project” [No. 202201010205] and “Guangdong Basic and Applied Basic Research Foundation” [No. 2022A1515110261]. The corresponding author had full access to all the data in the study and had final responsibility for the decision to submit for publication.

## Conflict of interest

The authors declare that the research was conducted in the absence of any commercial or financial relationships that could be construed as a potential conflict of interest.

## Publisher’s note

All claims expressed in this article are solely those of the authors and do not necessarily represent those of their affiliated organizations, or those of the publisher, the editors and the reviewers. Any product that may be evaluated in this article, or claim that may be made by its manufacturer, is not guaranteed or endorsed by the publisher.

## Abbreviations

EDs: Eating disorders
PHQ-9: Patient Health Questionnaire with 9 items
CHNS: the China Health and Nutrition Survey
CS: correlation stability
CBT-E: enhanced cognitive-behavioral therapy
