# Supplementary material for: Network analysis of eating disorder and depression symptoms among university students in the late stage of COVID-19 pandemic in China
Source: Front Nutr. 2023 May 25;10:1176076. doi: 10.3389/fnut.2023.1176076 (PMC10248072; doi:10.3389/fnut.2023.1176076)
Supplement: Supplementary file 1 [file Data_Sheet_1.docx]

**Supplementary Materials**

Gender differences of eating disorder and depression symptoms among Chinese university students in the later stage of the COVID-19 pandemic in China: a social network analysis


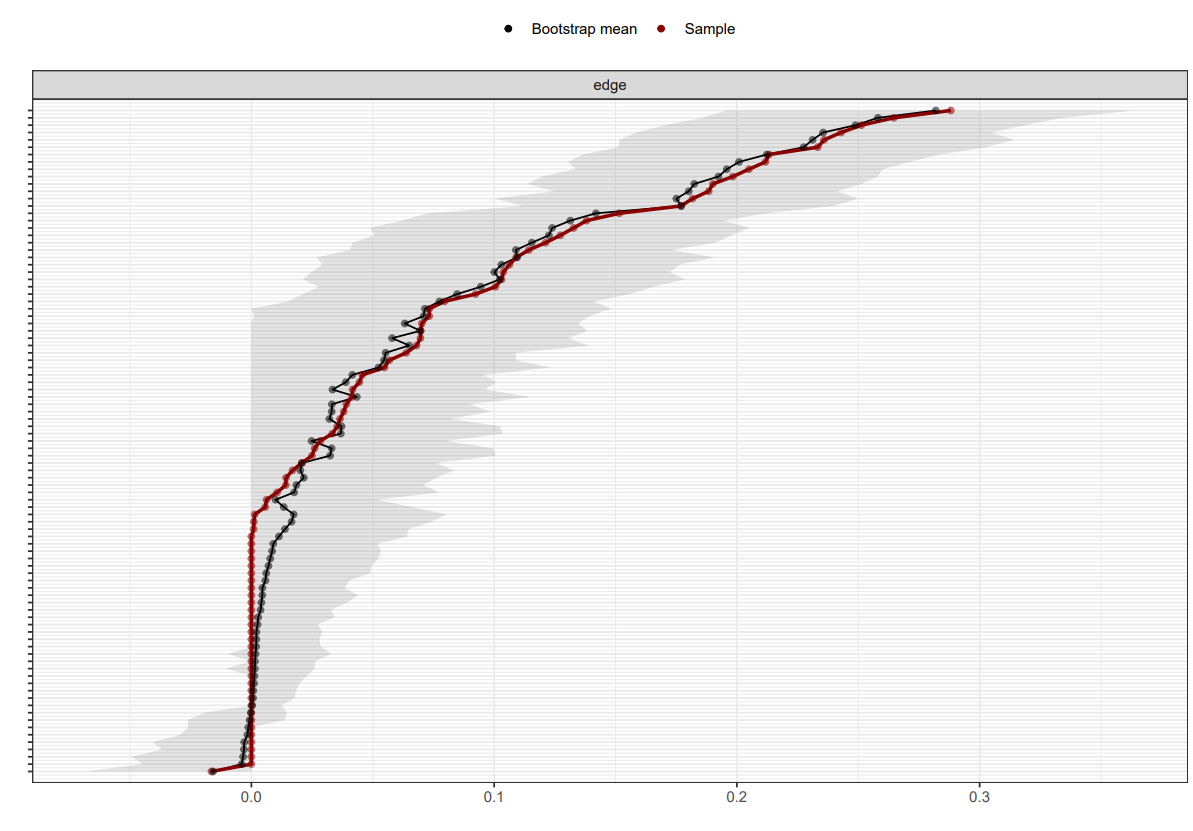


*Supplementary Figure 1.* Edge weight bootstrap of whole population.


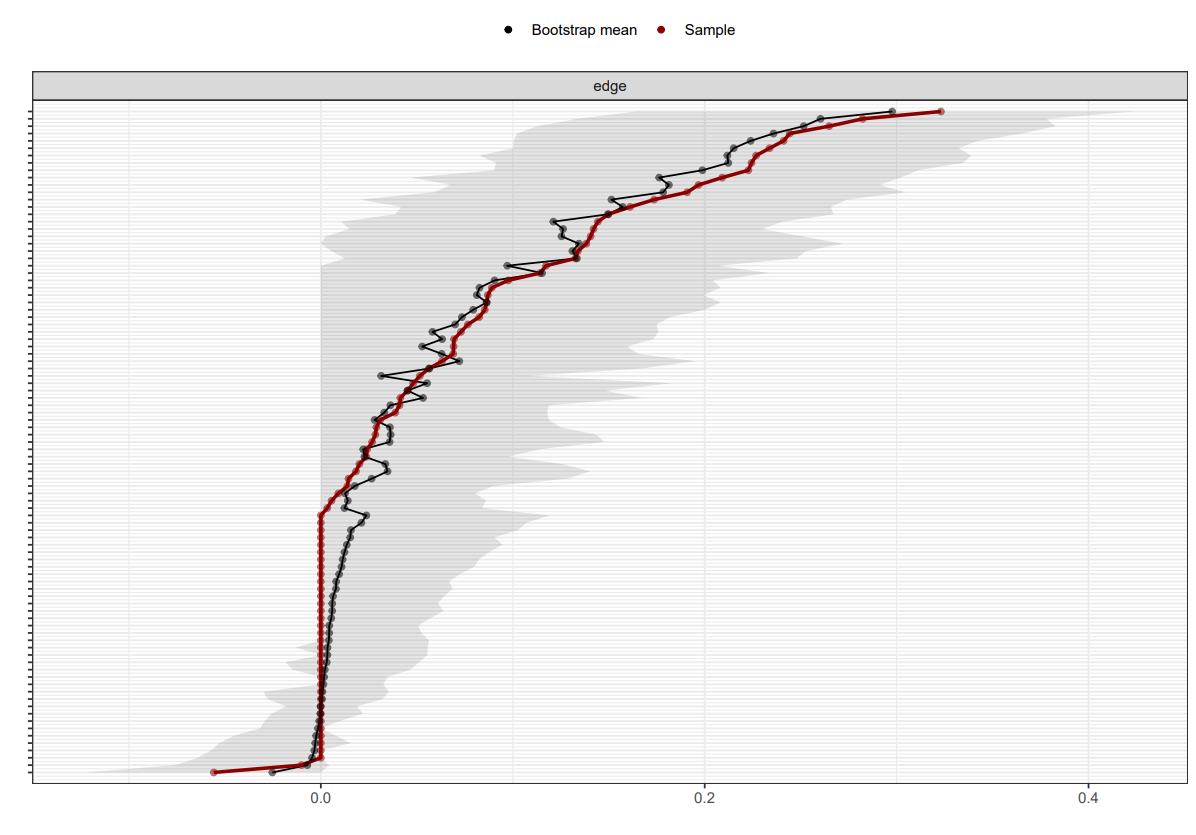


*Supplementary Figure 2.* Edge weight bootstrap of males.


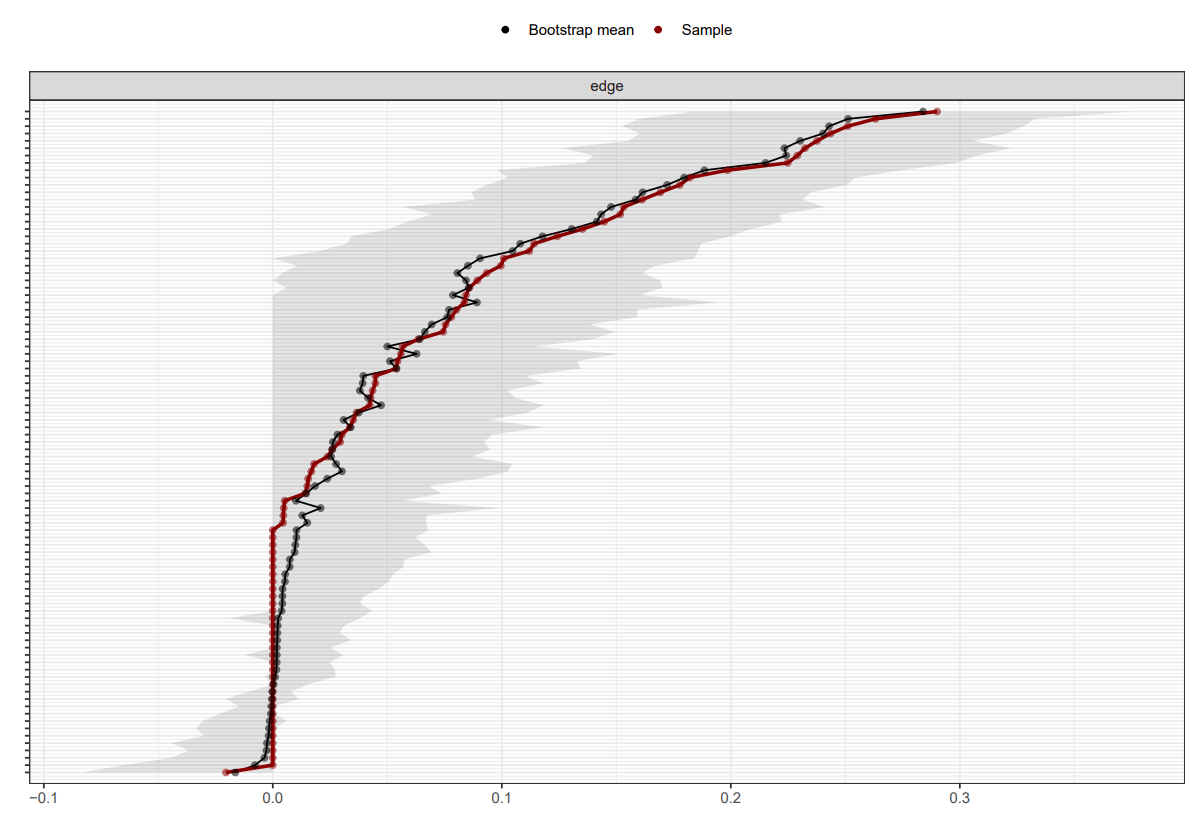


*Supplementary Figure 3.* Edge weight bootstrap of females.


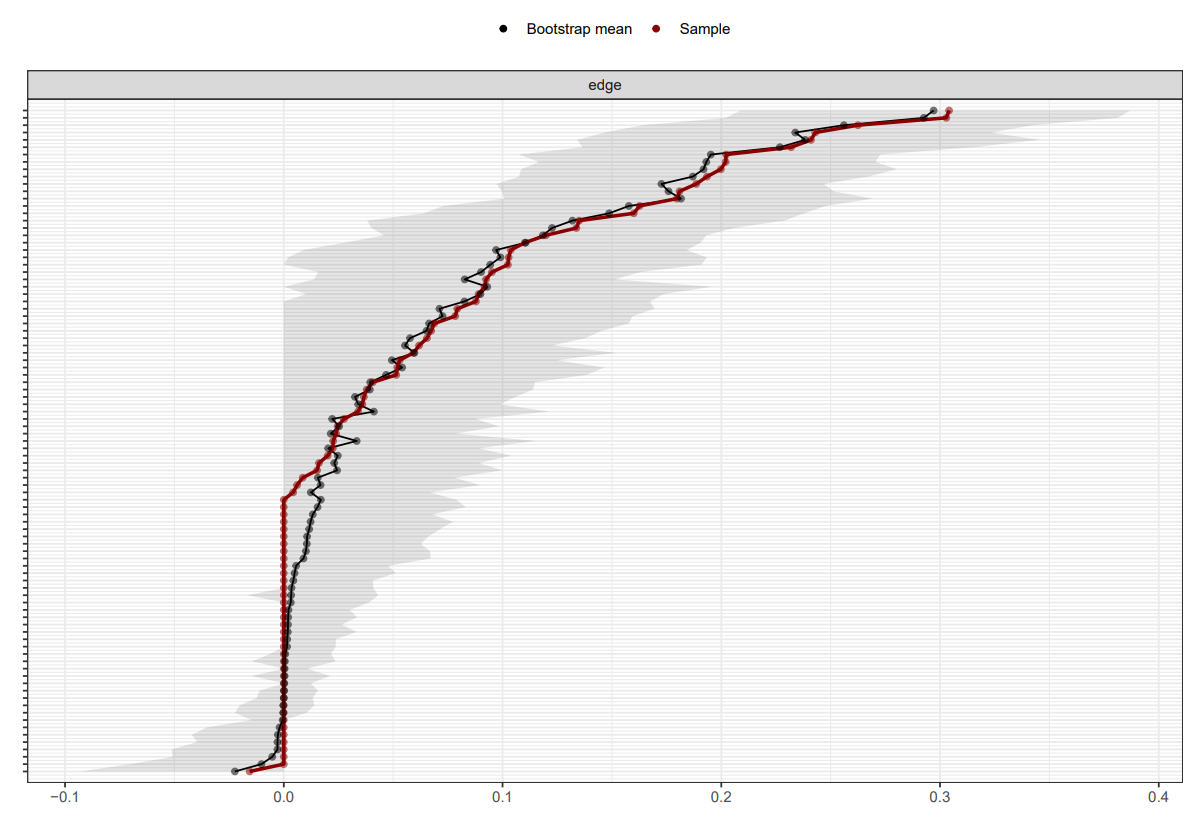


*Supplementary Figure 4.* Edge weight bootstrap of medical students.


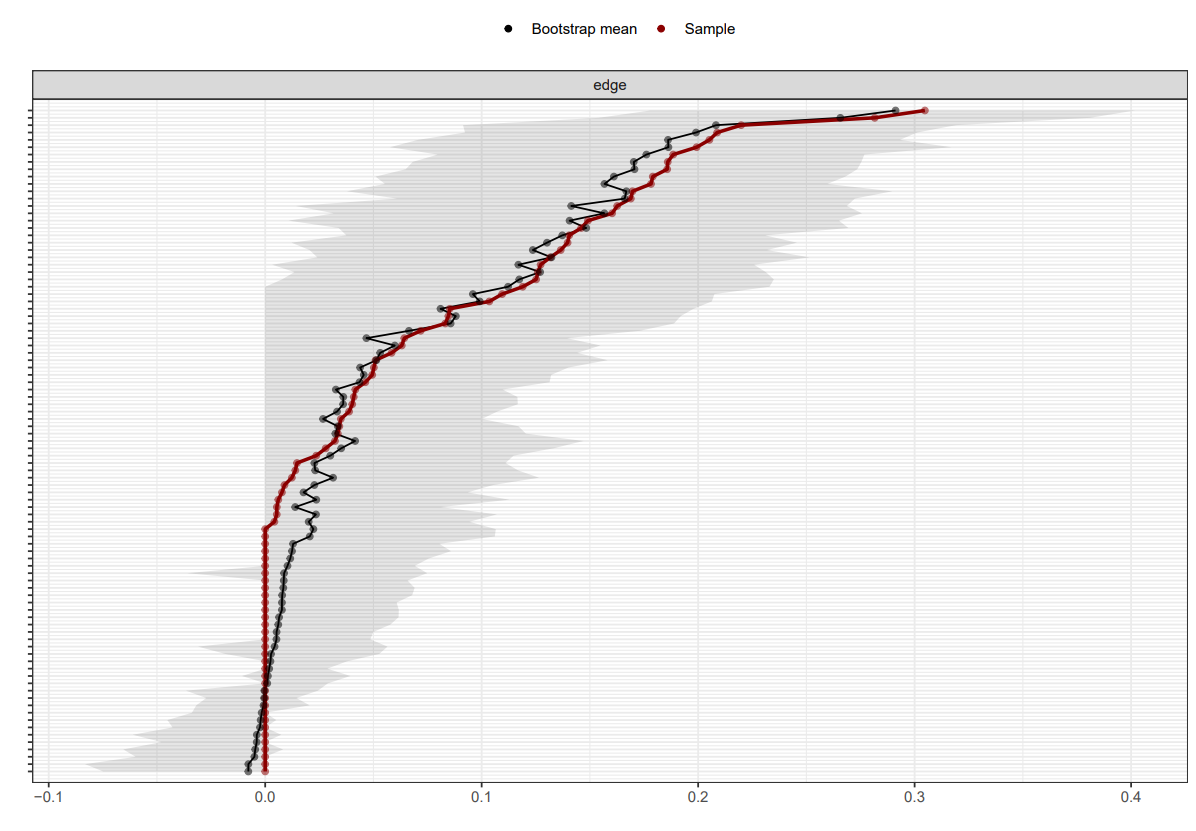


*Supplementary Figure 5.* Edge weight bootstrap of non-medical students.

*
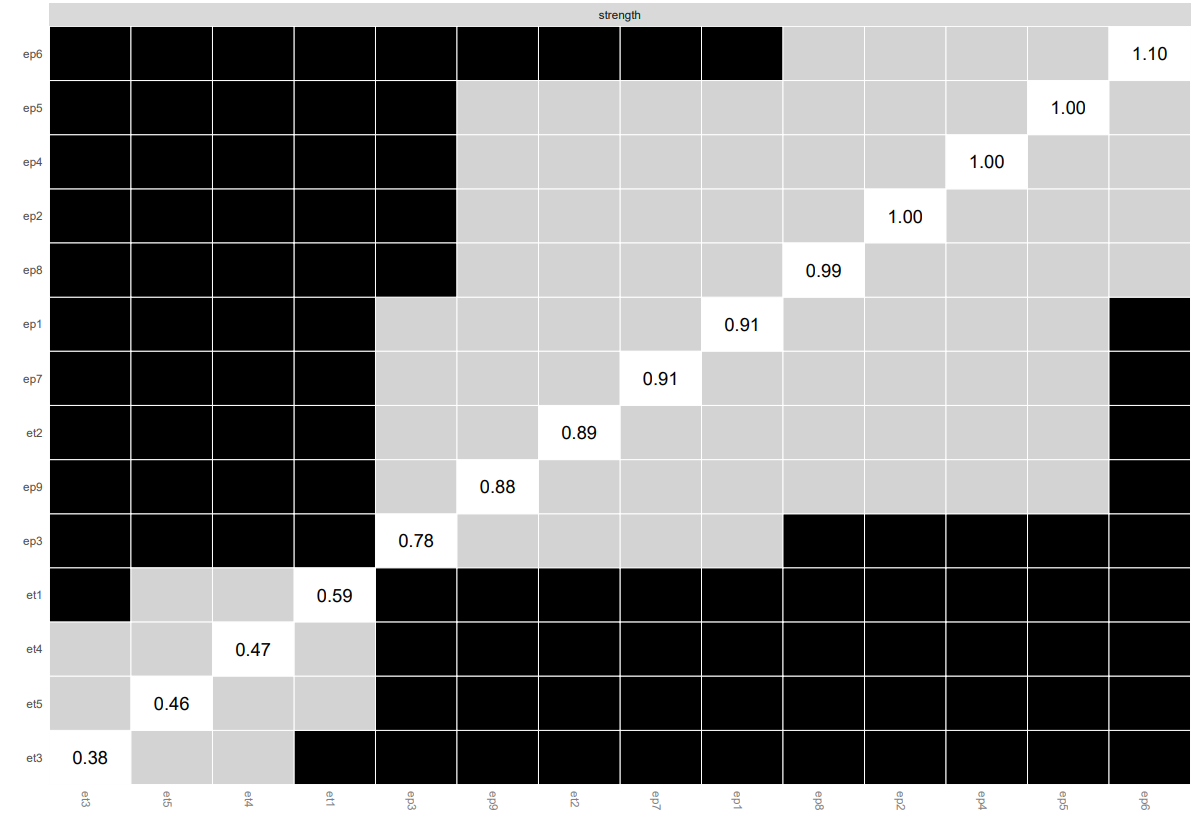
*

*Supplementary Figure 6.* Centrality difference of whole population.


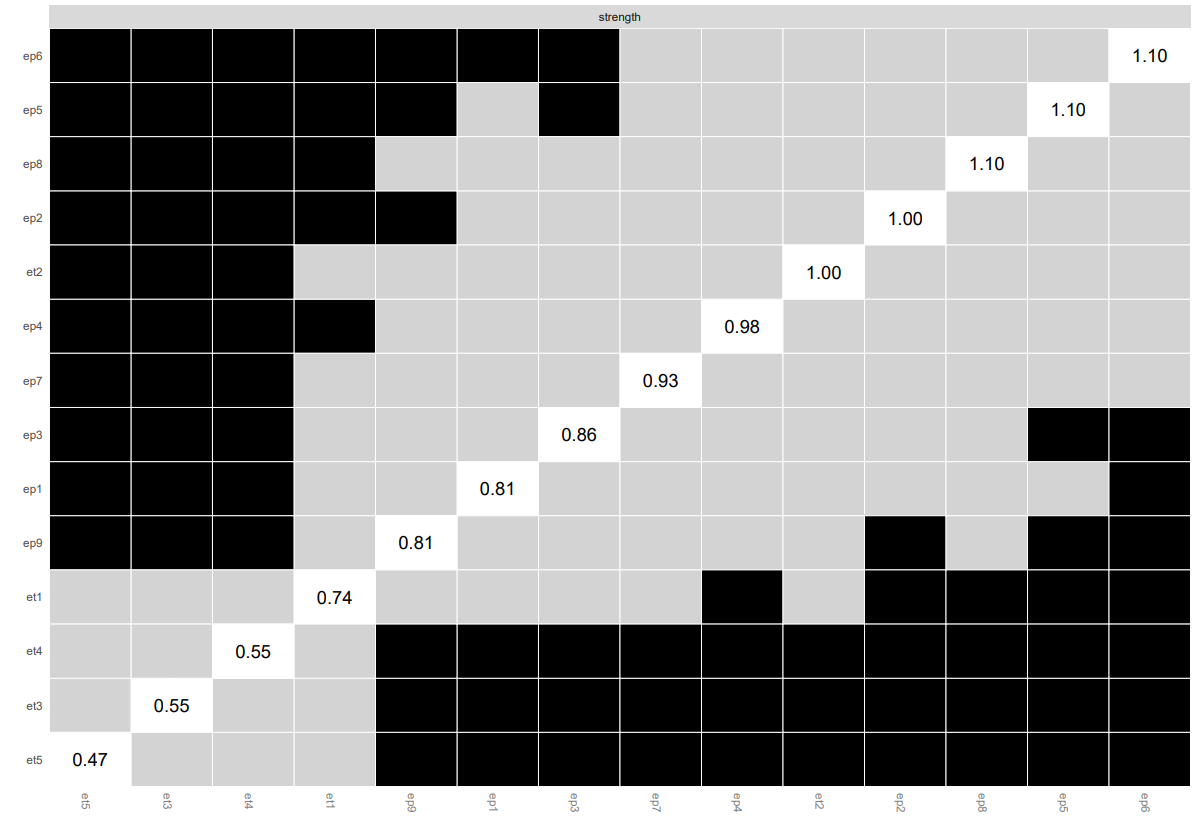


*Supplementary Figure 7.* Centrality difference of males.


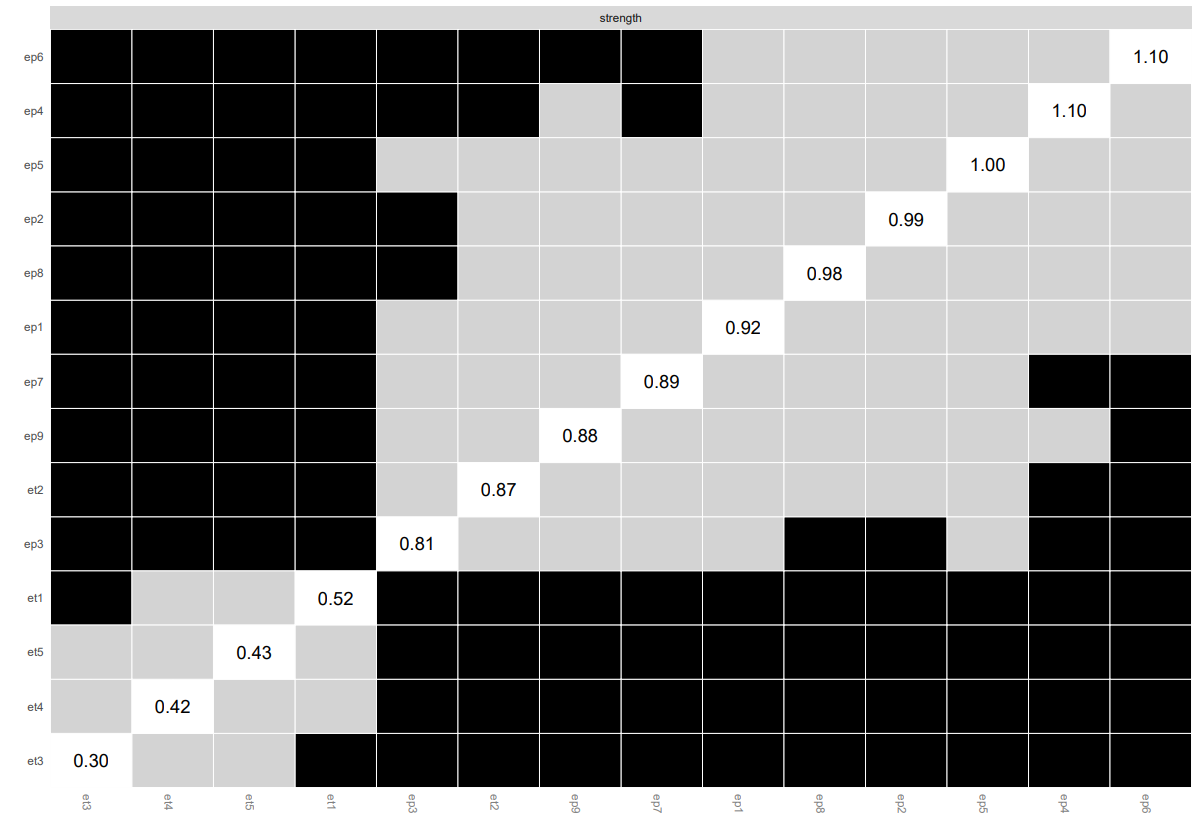


*Supplementary Figure 8.* Centrality difference of females.


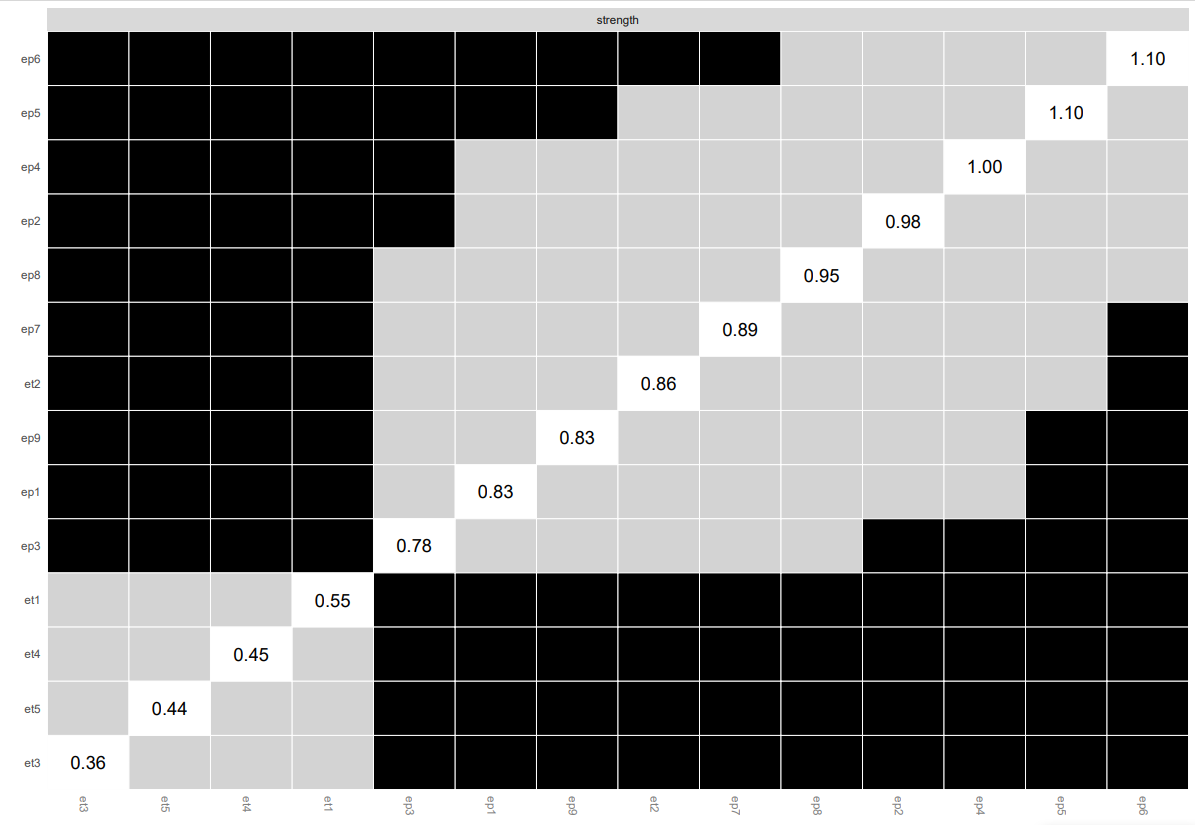


*Supplementary Figure 9.* Centrality difference of medical students.


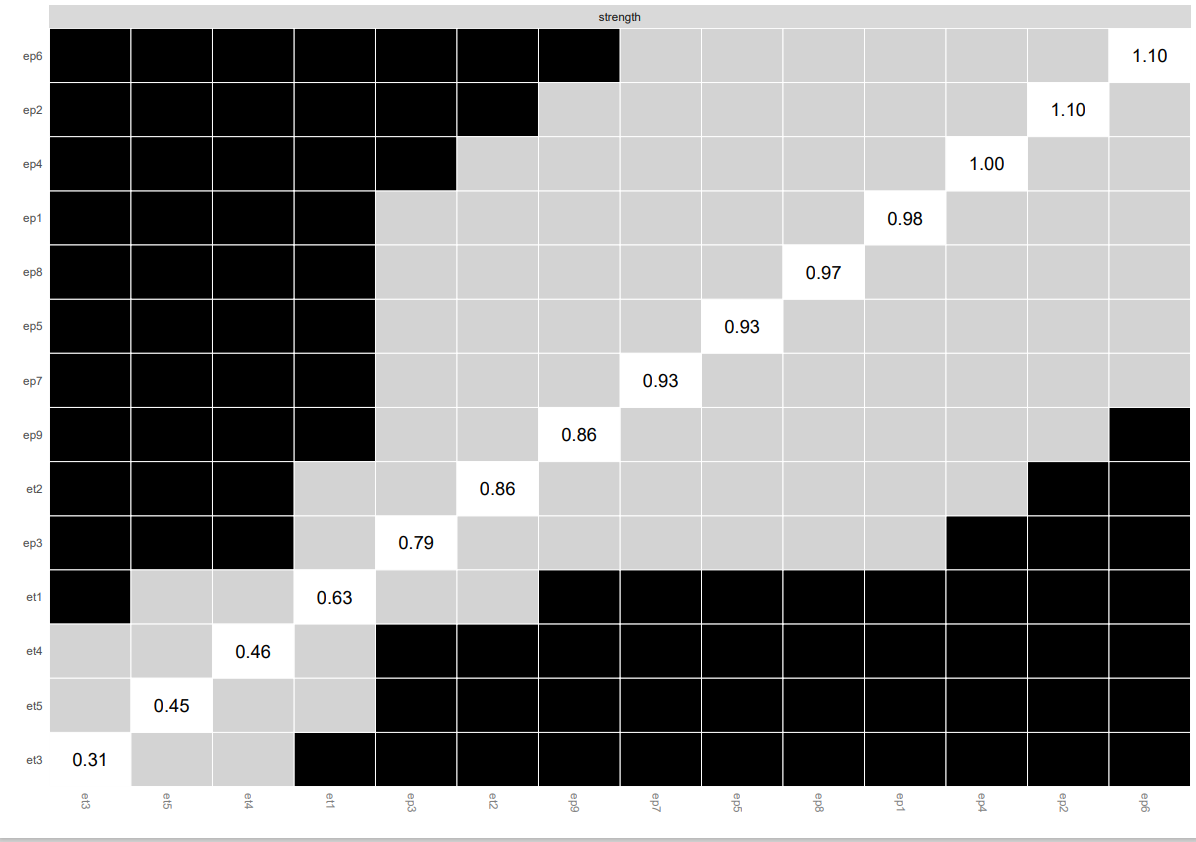


*Supplementary Figure 10.* Centrality difference of non-medical students.
